# Correlation Analysis of Vaginal Microbiome Changes and Bacterial Vaginosis Plus Vulvovaginal Candidiasis Mixed Vaginitis Prognosis

**DOI:** 10.3389/fcimb.2022.860589

**Published:** 2022-03-08

**Authors:** Bingbing Xiao, Disi A, Hanyu Qin, Lan Mi, Dai Zhang

**Affiliations:** Department of Obstetrics and Gynecology, Peking University First Hospital, Beijing, China

**Keywords:** mixed vaginitis, vulvovaginal candidiasis (VVC), vaginal microbiome, *Gardnerella*, *Atopobium*, *Lactobacillus*, bacterial vaginosis (BV)

## Abstract

Mixed vaginitis is the result of the simultaneous presence of different pathogenic processes mediated by at least two types of vaginal pathogens. Among the various types of mixed vaginitis presentations, bacterial vaginosis (BV) plus vulvovaginal candidiasis (VVC) presents to be the most prevalent form. Mixed vaginitis affects the health of women of all ages worldwide. However, few studies have focused on clinical manifestations, pathogenesis, diagnostic criteria, or therapy of mixed vaginitis. We recruited 48 symptomatic patients with clinical diagnoses of VVC complicated with BV, they were treated with oral metronidazole combined with local clotrimazole and followed to assess the drug efficacy and vaginal microbiome alterations before and after treatment. The vaginal microbiome in BV+VVC mixed vaginitis patients was altered significantly after the combined drug treatment within a unique form different from a simple overlay mode of BV and VVC, the key bacteria including *Gardnerella* and *Atopobium*, *Lactobacillus*. The combined drug therapy for the mixed vaginitis in this study was effective and enhanced treatment for BV may be more favorable because of more difficulty in dealing with BV according to the treatment outcome. The abundance of *Lactobacillus* in patients with mixed vaginitis affects the recovery of the vaginal microbiome as well as the prognosis, and the abundance should be actively restored. This is the first study to investigate the composition, diversity, and other characteristics of the vaginal microbiome in patients with BV+VVC mixed vaginitis before and after drug treatment, our results provide clues to improving the cure rate and reducing recurrences.

## Introduction

Mixed vaginitis, a syndrome combining symptoms of different pathogenic processes is mediated by at least two types of vaginal pathogens that cause vaginal inflammation. Common vaginitis includes bacterial vaginosis (BV), vaginal trichomoniasis (TV), vulvovaginal candidiasis (VVC), and aerobic vaginitis or desquamative inflammatory vaginitis (AV/DIV) (Paavonen and Brunham, 2018). Although some microorganisms always exhibit asymptomatic colonization, such as *Candida*, they are actually opportunistic pathogens and able to cause clinical symptoms when the immune system of the host is weakened. Mixed vaginitis presentations may be divided into various types such as BV+VVC, BV+TV, and AV+VVC according to their causative pathogenic processes, they require multiple therapies targeting all pathogens causing symptoms. Few studies on mixed vaginitis have focused on clinical manifestations, pathogenesis, diagnostic criteria and therapy. Mixed vaginitis needs to be distinguished from vaginal coinfections, which present clinical manifestations and signs caused by a single pathogen with asymptomatic concomitant colonization by another pathogen and, therefore, require treatments targeting only the pathogen causing symptoms (Sobel et al., 2013; Benyas and Sobel, 2022).

Attention has been drawn to the increasing prevalence of mixed vaginitis in recent years. According to a literature review summarizing mixed vaginitis advances, the proportion of symptomatic women with mixed vaginitis, most frequently presenting as genital itching, burning pain, and changes in the characteristics of discharge (odor, color, consistency), ranges from 4.44% to 35.06%; moreover, BV+VVC is the most prevalent form (Qi et al., 2021). During the past decade, the overall prevalence of mixed vaginitis in different regions of China varied greatly, fluctuating within a range from 7.33% to 41.87%, with a prevalence for the most common BV+VVC reaching 20.95% to 74.89% (Zhang and Liu, 2020). Regardless of the regional differences in feminine hygiene and the lack of global epidemiological studies, mixed vaginitis seems to have become much more common.

Even after considering mixed vaginitis as a separate vaginal infectious disease, many relevant facts remain unknown, especially the corresponding vaginal microbiome. The vaginal microbiome is characteristic of specific vaginitis types and determines both pathogenic and therapeutic outcomes; for instance, BV is a polymicrobial disorder of the vaginal microbiome that is characterized by the absence of vaginal lactobacilli (Paavonen and Brunham, 2018) and VVC by a *Candida* dominant vaginal microbiome (Kalia et al., 2020). Whether the vaginal microbiome of BV+VVC mixed vaginitis consists of a simple overlay microbiome or presents unique characteristics is unclear. For this study, we enrolled patients with BV+VVC mixed vaginitis and described their vaginal microbiome before and after treatment with oral metronidazole and local vaginal clotrimazole to provide evidence for improving the cure rate and reducing recurrences (van Schalkwyk and Yudin, 2015; Cooperative Group of Infectious Disease, 2021b; Workowski et al., 2021).

## Materials and Methods

### Study Design and Study Population

We selected record data from 48 18-to-50-year-old patients clinically diagnosed as having VVC complicated with BV, who were admitted to The First Hospital of Peking University from January 2017 to December 2020. BV was diagnosed using Amsel’s diagnostic criteria and identification of a Nugent score ≥7 from a vaginal Gram stain. Referring to the modified Amsel’s diagnostic criteria, BV was diagnosed when three of the following were present: a thin homogeneous discharge, elevated vaginal pH above 4.5, release of amines on the addition of 10% potassium hydroxide to vaginal fluid, and the presence of “clue” cells. VVC was diagnosed by identification of budding yeasts, hyphae, or pseudo-hyphae in a wet preparation (saline, 10% KOH) of vaginal discharge, or by identification of a culture or Gram staining yielding a positive result for a yeast species (Kalia et al., 2015; Xiao et al., 2016; Workowski et al., 2021). Two experienced microscopists blinded to the patients’ clinical information scored all the vaginal smears independently. The exclusion criteria included patients younger than 18 years or older than 50, *Trichomonas vaginalis* or AV/DIV, cervicitis or pelvic inflammation caused by *Neisseria gonorrhoeae* or *Chlamydia trachomatis*, pregnancy or lactation, menopause, innate or acquired immunodeficiency, and use of corticosteroid or antibiotics during the prior month.

No international medical standards for mixed vaginitis have been developed yet due to the lack of adequate studies. Referring to the latest version of *Sexually Transmitted Infections Treatment Guidelines, 2021* and the *Expert Consensus on Diagnosis and Treatment of Mixed vaginitis*, we adopted oral metronidazole combined with local clotrimazole to treat the BV+VVC mixed vaginitis (Cooperative Group of Infectious Disease, 2021a; Workowski et al., 2021). All patients were treated with metronidazole (400 mg orally twice per day for 7 days) combined with clotrimazole (500 mg intravaginally once per day on days 1, 4, and 7). We obtained informed consents from patients to perform follow-ups 7 ± 3 days after treatment termination; in addition, we also performed secondary follow-ups of patients with incomplete remission of symptoms after their second treatment. The follow-up observation indicators included a general examination and detailed anamnesis, a routine gynecological examination, a routine examination of discharge, vaginal discharge pH determination, Nugent scoring, and microecological detection of vaginal discharge.

Vaginal samples were collected before and after the drug treatment as well as at the time of follow-up. Two vaginal swabs were placed into the vagina at a standard anatomical site (one third of the lateral vaginal wall) and rubbed against the vaginal wall at each visit, one for microecological detection of vaginal secretions and the other for subsequent vaginal microbiota analysis. At every visit, a specimen was taken from each patient for a vaginal fungal culture and drug sensitivity test. To evaluate drug efficacy, we used fungal microscopic examinations for VVC (cured patients had negative results while the uncured ones had positive results) and Amsel’s diagnostic criteria combining Nugent scores for BV (scores between 0 and 3 identified cured patients, scores between 4 and 6 identified those with intermediate BV, scores between 7 and 10 identified uncured patients).

### 16S rRNA Gene Sequencing

Total genomic DNA was extracted from samples using the SDS method (Lim et al., 2016). 16S rRNA genes (V3-V4) were amplified using specific primers with a barcode. PCR products were mixed at equal ratios. Next, the PCR products mix was purified using a Qiagen Gel Extraction Kit (Qiagen, Germany). Sequencing libraries were generated using a TruSeq^®^ DNA PCR-Free Sample Preparation Kit (Illumina, USA) following manufacturer’s recommendations, and index codes were added. The library was sequenced on an Illumina NovaSeq using 2 x 250 base paired-end technology on an SP flow cell. This process generated an average of 93,000 reads per sample. Paired-end reads were merged using FLASH (VI.2.7, http://ccb.jhu.edu/software/FLASH/) (Magoč and Salzberg, 2011), and the splicing sequences were called raw tags. High quality tags were obtained (Bokulich et al., 2013) in a quality controlled process of QIIME (V1.9.1, http://qiime.org/scripts/splitlibrariesfastq.html) (Caporaso et al., 2010). The tags were compared with the reference database(Silva database, https://www.arb-silva.de/) using UCHIME algorithm (UCHIME Algorithm, http://www.drive5.com/usearch/manual/uchime_algo.html) (Edgar et al., 2011) to detect chimera sequences, and then the chimera sequences were removed (Haas et al., 2011). Then the Effective Tags finally obtained.

### UHPLC-MS/MS Analysis

UHPLC-MS/MS analyses were performed using a Vanquish UHPLC system (ThermoFisher, Germany) coupled with an Orbitrap Q ExactiveTM HF mass spectrometer (Thermo Fisher, Germany). Samples were injected onto a Hypesil Goldcolumn (100×2.1 mm, 1.9 μm) using a 17-min linear gradient at a flow rate of 0.2 mL/min. The Orbitrap Q ExactiveTM HF mass spectrometer was operated in positive/negative polarity modes. The raw data files generated by the UHPLC-MS/MS were processed using Compound Discoverer 3.1 (CD3.1, Thermo Fisher) to perform peak alignment, peak picking, and quantitation for each metabolite. Statistical analyses were performed using the statistical software R (R version R-3.4.3), Python (Python 2.7.6 version) and CentOS (CentOS release 6.6). We attempted normal transformations using an area normalization method for non-normally distributed data.

### Metabolite Analysis

The samples were placed into EP tubes and resuspended with pre-chilled 80% methanol using a vortex. Next, the samples were incubated on ice for 5 min and centrifuged at 15,000 *g* (4°C for 20 min). The supernatants were diluted to a final concentration containing 53% methanol by LC-MS grade water. The samples were subsequently transferred to fresh Eppendorf tubes and were then centrifuged at 15,000 *g*, 4°C for 20 min. Finally, the supernatants were injected into the LC-MS/MS system analysis (Want et al., 2006; Barri and Dragsted, 2013).

Metabolites were annotated using the KEGG (https://www.genome.jp/kegg/pathway.html), HMDB (https://hmdb.ca/metabolites) and LIPIDMaps (http://www.lipidmaps.org/) databases. Principal components analysis (PCA) and partial least squares discriminant analysis (PLS-DA) were performed with metaX (Wen et al., 2017) (a flexible and comprehensive software for processing metabolomics data). Volcano plots were used to filter metabolites of interest based on log_2_ (FoldChange), a log_10_ (*P*-value) of metabolites by ggplot2 in R language. For clustering heat maps, the data were normalized using z-scores of the intensity areas of differential metabolites and were plotted using a Pheatmap package in R language.

### Statistical Analysis

SPSS19.0 software was used for statistical analyses. We analyzed changes in vaginal pH and Nugent scores using *t* tests, the improvement of main symptoms and efficacy were analyzed by χ² and *U* tests (all conducted using bilateral tests). In addition to the main efficacy indicators, pairwise comparisons were not carried out between groups for other indicators, and *P*<0.05 indicated significant differences between groups. The description of quantitative indicators is a calculation of the mean, standard deviation, median, minimum, and maximum. Classification indicators and grade indicators are described in terms of the number and percentage of each category.

For community clustering analyses, the clustering of communities was based on community composition and abundance using complete linkage hierarchical clustering with four clusters using R (version 4.0.0) (Zhou et al., 2007). The PCoA and heatmap of main genus abundance figures of vaginal microbial communities were generated using the ade4 and ComplexHeatmap package in R (version 4.0.0). For alpha diversity analyses, the Shannon index at the operational taxonomic units (OTU) level was calculated with QIIME (Version 1.9.1). We used a Wilcoxon rank sum test for comparing Shannon index differences between groups. Marker vaginal bacteria analyses were performed using a differential abundance of bacteria by LDA Effect Size (Segata et al., 2011), and we used a *t*-test to compare bacteria abundances between groups. Only genera with LDA scores >3 and p<0.05 were regarded as significant. Statistical analyses were performed using R (version 4.0.0).

For marker metabolite analyses, we applied univariate analyses (*t*-tests) to calculate the statistical significance (*P*-value) between groups. The metabolites with VIP > 1 and *P*-values < 0.01 and log_2_ fold changes ≥1 or ≤ -1 were considered to be differential metabolites. Volcano plots were used to filter metabolites of interest based on log_2_ (FoldChange) and log_10_ (*P*-value) of metabolites by ggplot2 in R (version 4.0.0). Genera–metabolome correlations were obtained by computing pairwise correlation coefficients using Spearman’s correlation coefficients between marker genera and marker metabolites for each group. We focused on genera–metabolite pairs for which the correlation coefficients were greater than or equal to 0.4 (Benjamin–Hochberg adjusted *p <*0.05).

## Results

### Grouping Information and Clinical Characteristics of the Study Population

We enrolled 48 patients in this study. We obtained data from all patients before and after treatment and at follow-ups, 39 patients did not undergo recurrences (groups M1–M4 in **Table 1**) but 9 patients experienced recurrences (group MR in **Table 2**). Recurrence in our study was defined as that either of BV or VVC relapsed after drug treatment, while merely the recurrence of VVC was observed in our study.

**Table 1:** Grouping information of the study population without recurrences.

Group	VVC	BV	Total population (*n*)
M1	Cured	Cured	11
M2	Cured	Not cured	16
M3	Not cured	Not cured	9
M4	Not cured	Cured	3

**Table 2:** Specific information of the study population with recurrences.

Group	VVC	BV	Population (*n*)	Total population (*n*)
MR	Recurrent	Cured	6	9
		Not cured	3	

The mean age of the 48 patients was 32.92 ± 6.17 years; 38 patients had had previous VVC episodes (24 with a history of RVVC) and 13 patients had had previous BV episodes, while 9 patients had both. We observed that the symptoms and signs of patients with BV+VVC decreased significantly after treatment, indicating that this treatment plan can effectively relieve symptoms. The abundance of *Lactobacillus* and dominant bacteria in vaginal samples changed with the remission of symptoms. The vaginal pHs were lower than 4.5 after treatment (and significantly lower than the pHs before treatment).

### Analysis and Comparison of Microecology Between Groups Before and After Treatment

We analyzed the scores of clinical symptoms and signs before treatment, during follow-up and recurrence, as well as the results of microscopic microecological evaluations, and we found similar indicators before treatment and Nugent score decreases after treatment. Overall, the BV cure rate was only 47.9% (23/48) while that of VVC reached 56.3% (27/48), this significant difference (*p*<0.05) indicates that this treatment may not be sufficient against bacterial vaginosis. Further analysis of the BV treatment outcomes showed that Nugent scores increased in 4 individuals, remained unchanged in 9, and decreased in 35 individuals after treatment, suggesting that 72.9% of patients with BV presented improvement after treatment (**Supplementary Tables 1–3**).

All vaginal fungal cultures were *Candida albicans* sensitive to clotrimazole. The microecological indicators of the patients in the MR group after relapse did not change significantly compared to those after treatment, therefore, we could not identify the microecological changes that promoted fungal relapses at a microscopic level.

### Drug Treatment Affected the Composition of the Vaginal Microbiome

**Figure 1:**
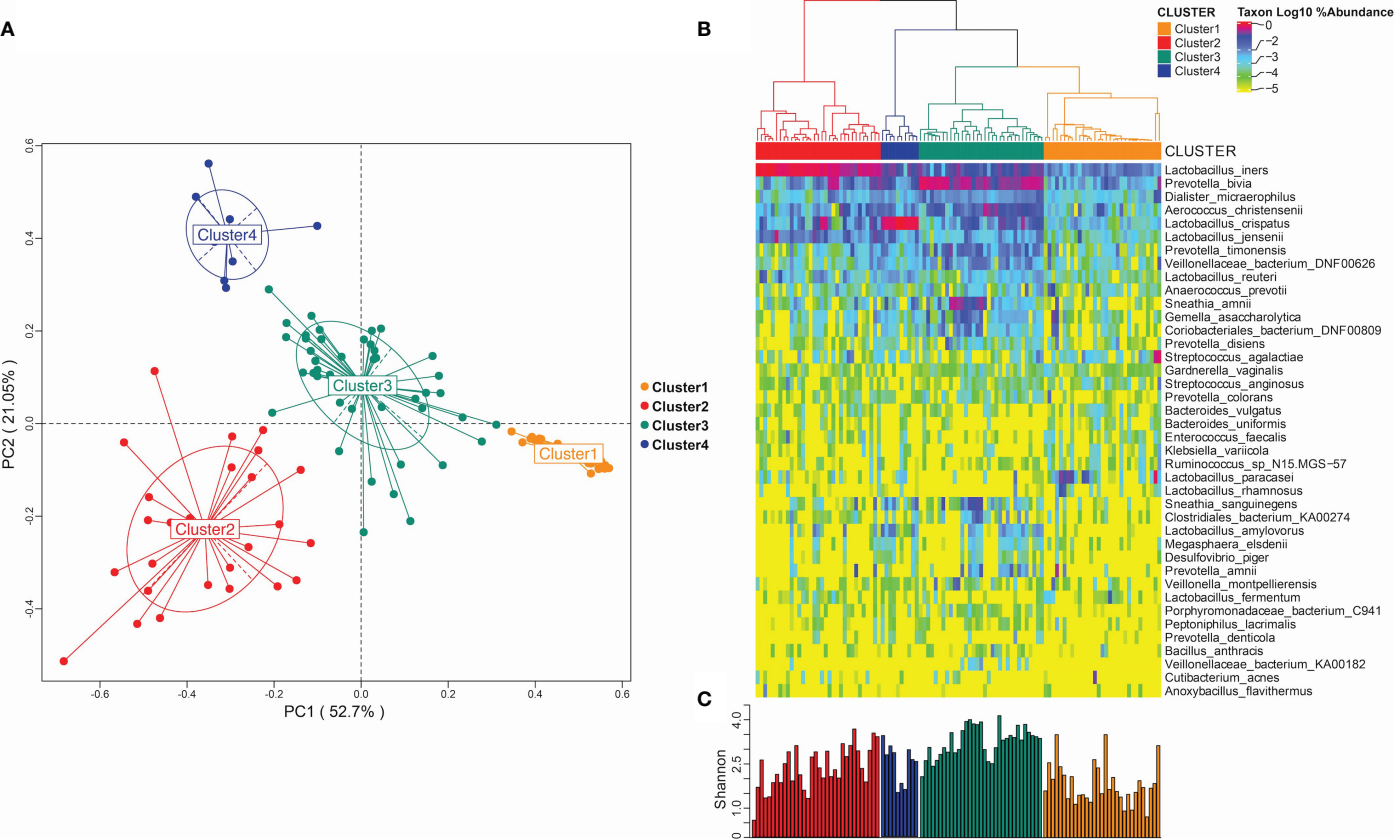
Microbiome of vaginal bacterial community. **(A)** Microbiome distribution obtained by PCoA clustering of the vaginal microbiota of all subjects; **(B)** heat maps of vaginal microbiome abundance distribution and the distribution of microbial community obtained by hierarchical clustering. **(C)** Shannon diversity index distribution in different microbial groups.

We classified the vaginal microbiome of all patients into clusters based on correlations between the members of their microbiome at the species level. As shown in **Figure 1A**, we divided each individual vaginal microbiome into four clusters with significant differences in their composition based on the species abundances at the species level. The heat map in **Figure 1B** shows the distribution of the horizontal abundance of each species in the four main clusters converted to log_10_. **Figure 1C** shows the bacterial Shannon diversity index distribution in different clusters. Among the four clusters, the highest diversity index was found in cluster 3 (**Figure 1C**, Cluster3), which was mainly enriched in *Prevotella* (**Figure 1B**, Cluster3). The Shannon diversity index of Cluster 1 was the lowest (**Figure 1C**, Cluster1), resulting in no enrichment of any single dominant genus (**Figure 1B**, Cluster1). By contrast, the Shannon diversity index of clusters 2 and 4 were higher than that of cluster 1 (**Figure 1C**, Cluster2, Cluster4), and both clusters were dominated by *Lactobacillus* microorganisms. Cluster 2 was mainly enriched in *Lactobacillus iners* (**Figure 1B**, Cluster2), while Cluster 4 was enriched in *Lactobacillus crispatus* (**Figure 1B**, Cluster4).

**Figure 2:**
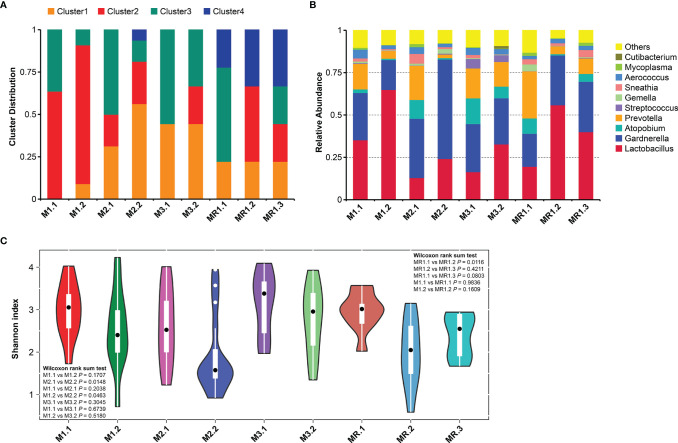
Changes in bacterial vaginal compositions in different patient groups. **(A)** Microbiota distributions before and after drug treatment. **(B)** Genus abundances before and after drug treatment. **(C)** Vaginal microbiome Shannon diversity indexes before and after drug treatment.

**Figure 2A** shows the vaginal microbiota distribution changes in different groups of patients after drug treatment. M1, M2 and M3 groups at different status were respectively represented as M1.1, M2.1, M3.1 (before treatment) and M1.2, M2.2, M3.2 (after treatment). MR group at different status were represented as MR.1 (before treatment), MR.2 (after treatment) and MR.3 (after recurrence). The microbiomes in patients of the M1 group were dominated by the genus Cluster2 and Cluster3 before treatment, while Cluster3 predominated in other groups of patients before the drug treatment (M2.1, M3.1, and MR.1). However, the vaginal microbiota changed significantly after the drug treatment and showed significant decreases in the proportions of Cluster3 in patient groups M1.2, M2.2, M3.2, and MR.2. In addition, the proportion of Cluster2 increased in patients of the M3 group after treatment (M3.2). By contrast, in the case of the patients of the relapse group (MR.1, MR.2, and MR.3), Cluster3 was predominant before treatment (MR. 1) and its proportion decreased or disappeared after treatment, while the proportions of Cluster2 and Cluster4 (dominated by *Lactobacillus*) increased significantly. However, after the relapse (MR.3), the proportion of Cluster 3 increased and the proportion of Cluster 2 decreased significantly.

**Figure 2B** shows the variation in vaginal microbial abundance distributions at the genus level among all groups of patients. We found significant vaginal microbial composition imbalances before drug treatment in all subgroups (M1.1, M2.1, M3.1, and MR.1) with low levels of *Lactobacillus* and high levels of BV-associated microorganisms, such as *Atopobium*, *Prevotella*, and *Gardnerella*. The vaginal microbiome genus distributions changed significantly after drug treatment. The abundance of *Lactobacillus* increased significantly while that of BV-related *Atopobium* and *Prevotella* decreased in all subjects after drug treatment. However, the abundance of *Gardnerella* decreased only in the M1 group (M1.2) and did not improve significantly in the other groups; in fact the abundance of *Gardnerella* increased in the M2 group (M2.1, M2.2).

### Drug Therapy Changes the Vaginal Microbiome Diversity

We conducted Shannon diversity analyses based on the distribution of at the vaginal species OTU for all patients, **Figure 2** shows the Shannon diversity index changes for different patient groups before and after drug treatment. The Shannon diversity results of all patients differed before and after drug treatment, and were mainly manifested by a decreased diversity of vaginal bacteria after drug treatment; in addition, we found a statistically significant difference between the results of the M2 group (**Figure 2C**, M2.1 *vs.* M2.2, *p*=0.0148) and those of the MR group (**Figure 2C**; MR.1 *vs*. MR.2; *p*=0.0116). By contrast, we found differences in the vaginal microbiota diversity in different groups of patients on the basis of their treatment outcomes. As shown in **Figure 2C**, the diversities of M1 group patients (M1.1 and M1.2) were higher than that of the M2 group patients (M2.1 and M2.2) before and after treatment. **Figure 2C** shows that the diversity of the M1 group patients (M1.1 and M1.2) was lower than that of the M3 group patients (M3.1 and M3.2) before and after treatment, but the difference was not statistically significant; in addition, the diversities of M3 group patients before and after treatment were similar (M3.1 *vs*. M3.2, *p*=0.3045). As shown in **Figure 2C**, the bacterial diversities of the MR group patients decreased significantly after treatment (MR.1 *vs*. MR.2, *p*=0.0116), but increased after relapse, and we found no significant differences when compared to those before treatment (MR.1 *vs*. MR.3, *p*=0.0803).

### Key Vaginal Microorganism Changes Associated With the Effect of Drug Therapy

**Figure 3:**
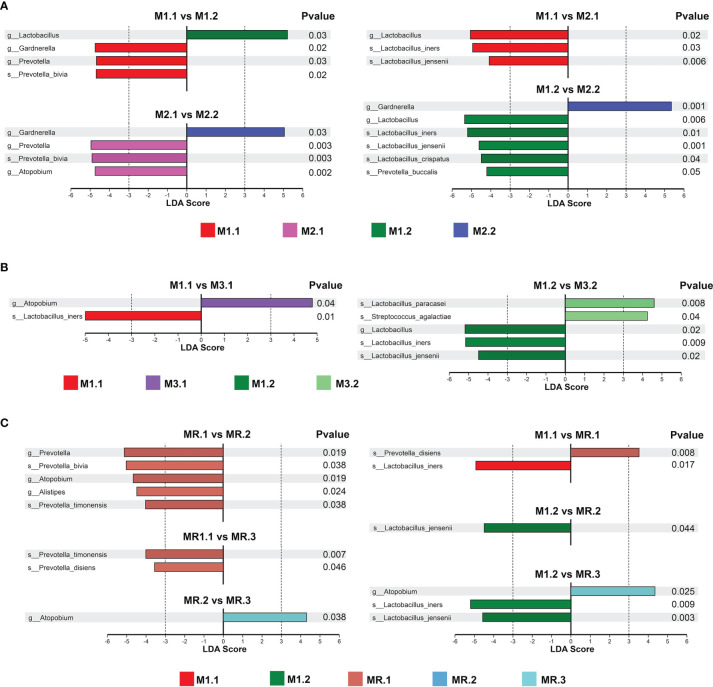
Key vaginal microorganism changes before and after treatment. LDA and T-test identified the most differentially abundant taxons between **(A)** patients completely cured (M1.1, M1.2) and patients with cured VVC plus uncured BV (M2.1, M2.2), **(B)** patients completely cured(M1.1, M1.2) and patients with cured BV plus uncured VVC (M3.1, M3.2), **(C)** patients completely cured (M1.1, M1.2) and patients with recurrence (MR.1, MR.2, MR.3).

We used linear discriminant analysis (LDA) and *t*-tests to analyze the changes in key vaginal microorganisms on the basis of different treatment outcomes and on the microbial abundance distributions at the genus and species levels for the patients’ groups (**Figure 3**).

As shown in **Figure 3A**, *Prevotella* and *Gardnerella* were predominant in M1.1 group patients before treatment, while *Lactobacillus* was predominant after treatment in M1.2 group patients. *Prevotella* and *Atopobium* were predominant in M2.1 group patients before treatment, while the *Prevotella* abundance decreased significantly after treatment in M2.2 group patients, but that of *Gardnerella* increased. Compared with the abundance in M2.1 group patients, the abundance of *Lactobacillus* was higher in M1.1 patients, suggesting that the failure to cure the BV may have been associated with the low baseline abundance of *Lactobacillus*. In addition, the abundance of *Lactobacillus*, especially those of *Lactobacillus iners*, *Lactobacillus jensenii* and *Lactobacillus crispatus*, was higher in M1.2 group patients than in M2.2 group patients with predominant *Gardnerella*. Moreover, the high abundance of *Gardnerella* probably correlates with the failure to cure BV.

As shown in **Figure 3B**, we found similar microbiome compositions in M3 group patients before and after treatment (M3.2 *vs*. M3.1), suggesting that the drug treatment failed to significantly change the vaginal microbiome of the patients in this group. The abundance of *Lactobacillus*, especially that of *Lactobacillus iners*, was lower in M3 group patients (M3.1 and M3.2) than in M1 group patients (M1.1 and M1.2) before and after treatment, suggesting a possible association between the treatment failure and a low *Lactobacillus* abundance. In addition, the abundance of *Atopobium* was high in M3 group patients before treatment (M3.1).

As shown in **Figure 3C**, in the recurrence group, the abundances of *Prevotella* and *Atopobium* were significantly decreased after treatment (MR.2) compared with those before treatment (MR.1); however, after the recurrence (MR.3), the abundance of *Atopobium* increased to a level similar to that before treatment (MR.1). By contrast, patients in the MR group (MR.1, MR.2 and MR.3) had lower *Lactobacillus* abundances (especially lower *Lactobacillus iners* and *Lactobacillus jensenii* abundances) and a higher *Atopobium* abundance than patients in the M1 group (M1.1 and M1.2) before and after treatment.

### Correlation Between Metabolites and Key Vaginal Microbiota Associated With the Therapy Outcomes

**Figure 4:**
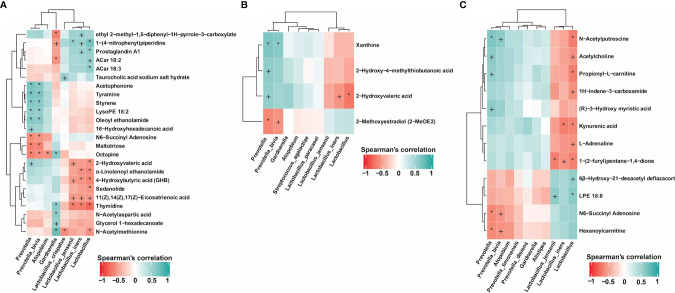
Correlation between metabolites and key vaginal microbiota associated with the drug therapy outcomes. **(A)** Association of key vaginal microorganisms and metabolites in patients completely cured (M1.1, M1.2) and in patients with cured VVC and untreated BV (M2.1, M2.2). **(B)** Associations between key vaginal microorganisms and metabolites in completely cured patients (M1.1, M1.2) and in uncured patients (M3.1, M3.2). **(C)** Association of key vaginal microorganisms and metabolites in completely cured patients (M1.1, M1.2) and in patients with recurrence (MR.1, MR.2, MR.3). P value of Spearman’s correlation: ^+^<0.05, *<0.01.

The key bacteria found to be associated with the treatment outcomes included *Lactobacillus*, *Atopobium*, *Prevotella* and *Gardnerella*. In addition, we identified metabolites that were differentially expressed in vaginal discharge of different patients, including 2-hydroxyvaleric acid, tyramine, acetophenone, styrene, LysoPE, oleoyl ethanolamide, 16−hydroxyhexadecanoic acid, N−acetylmethionine, propionyl−L−carnitine, glycerol 1−hexadecanoate, α -linolenoyl ethanolamide, 4−hydroxybutyric acid (GHB), sedanolide, and thymidine (**Supplementary Figures 1–5**). We conducted correlation analyses to assess the potential association between key vaginal bacteria (*Lactobacillus*, *Atopobium*, *Prevotella* and *Gardnerella*) associated with the treatment outcomes and the differential metabolites in vaginal secretions (**Figure 4**).

As shown in **Figure 4A**, in the patients completely cured (M1.1 and M1.2) and in those with cured VVC and untreated BV (M2.1 and M2.2), the association of differential metabolites with the presence of key vaginal microorganisms showed that metabolites including tyramine, acetophenone, styrene, LysoPE, oleoyl ethanolamide, 16− hydroxyhexanoic acid, 2−hydroxyvaleric acid, thymidine, glycerol 1−hexadecanoate, N−acetylaspartic acid, and N−acetylmethionine were positively correlated with the presence of BV-related microorganisms including *Atopobium*, *Prevotella* and *Gardnerella*. In addition, 2−hydroxyvaleric acid, α-linolenoyl ethanolamide, 4−hydroxybutyric acid (GHB), sedanolide, thymidine, and N−acetylmethionine were negatively correlated with the presence of *Lactobacillus*. Moreover, 2-hydroxyvaleric acid was not only positively correlated with the presence of *Prevotella* but also negatively correlated with *Lactobacillus* and *Lactobacillus iners*. By contrast, we found amine metabolites, benzoones and acids such as tyramine, acetophenone, 16−hydroxyhexadecanoic acid, and 2−hydroxyvaleric acid to be positively correlated with the presence of *Prevotella*. Thymidine and N−acetylmethionine were also positively correlated with the presence of *Gardnerella* and negatively correlated with the presence of *Lactobacillus*, especially of *Lactobacillus crispatus*. *Gardnerella* was the predominant microorganism after treatment in the patients with cured VVC and untreated BV.

In completely cured patients (M1.1 and M1.2) and in uncured patients (M3.1, M3.2), the association analysis of differential metabolites with key vaginal microorganisms (**Figure 4B**) showed that 2−hydroxyvaleric acid was positively correlated with the presence of *Prevotella* and negatively correlated with the presence of *Lactobacillus* and *Lactobacillus iners*, further illustrating the important role of 2−hydroxyvaleric acid in the treatment of BV and VVC. By contrast, in completely cured patients (M1.1 and M1.2) and in patients with recurrences (MR.1, MR.2, and MR.3), our association analysis of differential metabolites and differential species revealed positive correlations between N−acetylputrescine, acetylcholine, and propionyl−L−carnitine and the presence of *Prevotella* and negative correlations with the presence of *Lactobacillus* (**Figure 4C**).

## Discussion

For this study, we investigated the composition, diversity and other characteristics of the vaginal microbiome in patients with BV+VVC mixed vaginitis before and after drug treatment. As mentioned, mixed vaginitis and vaginal co-infections are different entities. Based on the literature and our own results, patients presenting BV and VVC simultaneously have their own characteristic symptoms and signs of vaginitis rather than a simple overlay microbiome, *Lactobacillus iners* and *Prevotella* predominate in their vaginal microbiome and the abundances of *Gardnerella* and *Atopobium* are also higher than those in healthy women. Previous studies have focused on single types of vaginitis or in asymptomatic co-infections with multiple pathogenic micro-organisms. For example, in 2005, Fredricks et al. (Fredricks et al., 2005) found that compared with the vaginal microbiome of healthy women, that of women with BV had a significant increase in its diversity, with low abundance of *Lactobacillus crispatus* (significantly decreased to almost inexistent) but detection of *Lactobacillus iners* in most patients. In addition, the abundances of *Gardnerella*, *Atopobium*, *Megasphaera*, *Leptotrichia*, and BVAB 1-3 were significantly increased and correlated with the presence of BV (*p <*0.001). McKloud et al. (McKloud et al., 2021) found that the vaginal microbiome of women with recurrent VVC was dominated by bacteria of the genus *Lactobacillus* with a significant increase in the *Candida* abundance compared with the abundance in healthy women. The levels of bacterial diversity and the abundances of vaginal anaerobic bacteria such as *Gardnerella* and *Prevotella* were similar, but slightly higher in healthy women. The abundances of *Lactobacillus crispatus* and *Lactobacillus jensenii* dropped from 44% in healthy women to 30% in women with RVVC, while the abundance of *Lactobacillus iners* increased from 19% in healthy women to 40% in women with RVVC. The abundances of *Lactobacillus iners* in the two groups were significantly different.

On the basis of the high abundances of BV-related pathogens in the treatment failure and recurrence groups in this study, we believe that BV-related pathogens play a major pathogenic role in patients with BV+VVC mixed vaginitis and determine their treatment outcome and the occurrence of relapses. We found that *Gardnerella* abundance increased in the VVC cured but BV uncured group after treatment (M2.2); patients in the MR recurrence group had lower *Lactobacillus* abundances (especially *Lactobacillus iners* and *Lactobacillus jensenii*) and a higher *Prevotella and Atopobium* abundance than patients in the M1 group before and after treatment*. Gardnerella*, *Prevotella*, and *Atopobium* were the main pathogenic bacteria of BV in our study, and other studies have identified synergistic effects between them, allowing them to form multi-strain biofilms and to further lead to refractory infections and recurrences. Moreover, each bacterium plays a different role during the infections. *Prevotella* can secrete proteases to degrade host antibodies and transfer ammonia to *Gardnerella*, leading to excessive secretion of ammonia in the host vagina, reducing mucosal immunity (Xie et al., 2020), and promoting the growth of other anaerobic bacteria. However, in the course of our limited follow-up, we found that the abundance of *Prevotella* decreased significantly after metronidazole treatment and did not increase again, a finding consistent with the reported high drug sensitivity of *Prevotella* to metronidazole (Ulger Toprak et al., 2018). Thus, we believe that *Prevotella* probably did not play a significant role in recurrences. *Gardnerella*, the main bacteria in the formation of BV biofilms, can adhere to vaginal epithelial cells and acts as a scaffold for the formation of biofilms. Dense biofilms can tolerate high concentrations of H_2_O_2_ and lactic acid, and have enhanced resistance to antibiotics and host mucosal immune defenses (Hardy et al., 2017). The presence of this specific type of biofilm can lead to resistance of *Gardnerella* to metronidazole, causing BV treatment difficulties and recurrences (He et al., 2021). In the BV pathogenesis model (Muzny et al., 2020), *Gardnerella* and *Prevotella*, as initially colonizing bacteria, are highly abundant in the vagina of patients with BV, but the formation of a biofilm does not induce a strong inflammatory response in vaginal epithelial cells and instead promotes immune escape. The secondary colonizer *Atopobium* is more likely to effectively stimulate the host to produce a strong immune response against the BV, leading to the corresponding signs and symptoms. A synergistic effect may exist between the two types of colonizers (Castro et al., 2019; Vestby et al., 2020). In addition, previous studies have found that the sensitivity of different clinical isolates of *Atopobium* to metronidazole varies greatly and more than half of the isolates display drug resistance to metronidazole (Schuyler et al., 2015; Shaskolskiy et al., 2016; Mendling et al., 2019), which suggests that targeted drugs with high sensitivity to *Atopobium* are needed to reduce recurrences during the treatment of patients with BV+VVC mixed vaginitis.

Although multi-species biofilms play a role in recurrences, the type and abundance of *Lactobacillus* also had a considerable influence on infection and recurrences (Ceccarani et al., 2019; Redelinghuys et al., 2020). The results of this study showed that before treatment the completely cured patients (M1.1) had a higher abundance of *Lactobacillus iners* than the patients with recurrences (MR.1), whereas the significantly higher abundance of *Lactobacillus jensenii* was established earlier in the completely cured patients after treatment (M1.2) than in the patients with recurrences (MR.2), suggesting that the abundance of *Lactobacillus iners* and *Lactobacillus jensenii* correlate with the recurrence of BV+VVC mixed vaginitis after a cure. On the basis of existing findings and our results, we can speculate that the absence of *Lactobacillus crispatus* and other protective *Lactobacillus* from the vaginal microenvironment of patients with mixed vaginitis, *Lactobacillus iners* may tend to act as the protector of the vaginal epithelium. Verwijs et al. (Verwijs et al., 2020) assessed the vaginal microbiota of 68 women with BV who received metronidazole treatment and found results similar to ours: *Lactobacillus iners* was the most common *Lactobacillus* species before and after treatment, the abundance of *L. jensenii* was also significantly increased after treatment, and that of *Lactobacillus crispatus* was significantly decreased before treatment and only slightly increased after treatment. After analyzing the vaginal microbiota of patients treated with metronidazole gel, *Lactobacillus iners* was the only dominant strain 30 days after treatment, indicating that *Lactobacillus iners* was resistant to metronidazole, while *Lactobacillus crispatus* was probably more susceptible to metronidazole in the BV environment (Petrova et al., 2017). Moreover, due to the ineffectiveness of metronidazole against *Lactobacillus iners*, the removal of BV-related pathogens with therapy results in a vaginal microbiome with predominant *Lactobacillus iners*. Therefore, combining with our results, assessing the level of *Lactobacillus iners* in the vagina of patients with mixed vaginitis before treatment may have a predictive value for the treatment outcome and, promoting the recovery of the *Lactobacillus* abundance in the vagina may help to enhance the treatment effects beyond antibiotic treatment.

This study was limited mainly by the size of the patient cohort, and larger prospective studies are needed to confirm our results. This is our first study to explore the vaginal microbiome of the patients with BV+VVC mixed vaginitis, so we merely measured the relative abundances. Apart from this, all our fungal culture results showed *Candida albicans*, preventing us from studying more on various vaginal fungus. In addition, the final cure rates of BV and VVC in this study were 47.92% (23/48) and 56.25% (27/48), respectively, revealing a better therapeutic effect for VVC than for BV. The cure rates in our study were low probably owing to our therapy targeting VVC with more potency than BV, future studies should apply longer periods of BV-targeted treatments.

In conclusion, we observed significant vaginal microbiome alterations in patients with BV+VVC mixed vaginitis before and after drug treatment, with the key causative bacteria including *Gardnerella*, *Atopobium*, and *Lactobacillus*. We have found some special bacterial in this study and we will consider proceeding with further quantitative research in the subsequent studies. The therapy of oral metronidazole with local clotrimazole to treat BV+VVC mixed vaginitis was effective in our study. And to enhance treatment for BV may be more favorable for prognosis of patients with BV+VVC mixed vaginitis. The abundance of *Lactobacillus* in women with mixed vaginitis has a great influence on the recovery of a normal vaginal microbiome and on the prognosis, and it should be actively restored.

## Data Availability Statement

The datasets presented in this study can be found in online repositories. The names of the repository/repositories and accession number(s) can be found below: NCBI SRA database, accession PRJNA801439 and EMBL-EBI MetaboLights database, accession MTBLS4222.

## Ethics Statement

The studies involving human participants were reviewed and approved by The Ethics Committee of Peking University First Hospital (V2.0/201504.20). The patients/participants provided their written informed consent to participate in this study. Written informed consent was obtained from the individual(s) for the publication of any potentially identifiable images or data included in this article.

## Author Contributions

BX and DZ conceived the study design. BX, LM, and DZ recruited volunteers and collected samples. LM and DZ were responsible for performing the laboratory assays. BX, DA, and HQ performed the data analysis. DA wrote the initial manuscript. BX and DZ revised the manuscript. All authors contributed to the article and approved the submitted version.

## Funding

This work was supported by the grants of the National Natural Science Foundation of China (No. 81971342 and No. 81200411).

## Conflict of Interest

The authors declare that the research was conducted in the absence of any commercial or financial relationships that could be construed as a potential conflict of interest.

## Publisher’s Note

All claims expressed in this article are solely those of the authors and do not necessarily represent those of their affiliated organizations, or those of the publisher, the editors and the reviewers. Any product that may be evaluated in this article, or claim that may be made by its manufacturer, is not guaranteed or endorsed by the publisher.
