# Supplementary material for: Correlation Analysis of Vaginal Microbiome Changes and Bacterial Vaginosis Plus Vulvovaginal Candidiasis Mixed Vaginitis Prognosis
Source: Front Cell Infect Microbiol. 2022 Mar 8;12:860589. doi: 10.3389/fcimb.2022.860589 (PMC8970117; doi:10.3389/fcimb.2022.860589)
Supplement: Supplementary file 1 [file DataSheet_1.docx]

**Supplementary Table 1** General characteristics of the study population before and after drug treatment

| Classifications | Before treatment | After treatment | statistics | *p* |
| --- | --- | --- | --- | --- |
| Amsel’s diagnostic criteria | 5.21±2.31 | 1.29±1.66 | t=9.520 | 0.000 |
| Nugent score | 6.69±1.65 | 3.83±2.67 | t=6.300 | 0.000 |
| AV score | 2.85±0.751 | 1.98±1.35 | t=3.890 | 0.000 |
| pH | 4.41±0.46 | 4.15±0.40 | t=2.935 | 0.004 |
| Cleanliness (I/II/III) | 27/15/6  (56.3/31.3/12.5%) | 32/10/6  (66.7/20.8/12.5%) | Fisher=1.449 | 0.491 |
| leukocyte counts (<10/hp vs ≥10/hp) | 36/12 (75.0/25.0%) | 42/6（87.5/12.5%） | χ2=2.462 | 0.17 |
| Classification of *Lactobacillus* (I/IIa/IIb/III) | 0/0/26/21  (0/0/55.3/44.7%) | 3/12/19/14  (6.3/25.0/39.6/29.2/%) | Fisher=18.756 | 0.001 |
| *Trichomonas* (+) | 0 | 0 |  |  |
| Fungus (+) | 48 | 12 | χ2=757.600 | 0.000 |
| BV (Negative/intermediate/typical) | 0/18/30  (0/37.5/62.5%) | 20/19/9  (41.7/39.6/18.8%) | χ2=31.335 | 0.000 |

**Supplementary Table 2** Characteristics of the study population in different groups before and after drug treatment

| Classifications | Group | Before treatment | After treatment | statistics | *p* |
| --- | --- | --- | --- | --- | --- |
| Age | M1 | 33.82±5.62 |  | F=0.676 | 0.612 |
|  | M2 | 32.31±5.83 |  |  |  |
|  | M3 | 34.00±5.20 |  |  |  |
|  | M4 | 36.33±3.06 |  |  |  |
|  | MR | 30.67±8.83 |  |  |  |
| Medical history (Neither/VVC/BV/Both) | M1 | 1/9/1/0 |  | Fisher=9.737 | 0.616 |
|  | M2 | 3/9/2/2 |  |  |  |
|  | M3 | 1/4/0/4 |  |  |  |
|  | M4 | 0/2/0/1 |  |  |  |
|  | MR | 1/5/1/2 |  |  |  |
| Amsel’s diagnostic criteria | M1 | 6.09±2.21 | 0.45±1.04 | Before treatment F=0.562 | 0.692 |
|  | M2 | 5.06±2.79 | 1.19±1.28 | After treatment F=2.955 | 0.029* |
|  | M3 | 5.00±2.40 | 2.78±2.33 |  |  |
|  | M4 | 4.33±2.08 | 1.00±1.00 |  |  |
|  | MR | 4.89±1.54 | 1.11±1.70 |  |  |
| Nugent score | M1 | 6.55±1.57 | 1.27±1.10 | Before treatment F=0.593 | 0.670 |
|  | M2 | 6.81±1.47 | 5.81±1.80 | After treatment F=14.997 | 0.000* |
|  | M3 | 6.89±1.69 | 5.56±1.42 |  |  |
|  | M4 | 5.33±2.31 | 1.33±1.53 |  |  |
|  | MR | 6.89±1.97 | 2.56±2.70 |  |  |
| AV score | M1 | 3.00±1.05 | 0.82±1.17 | Before treatment F=0.270 | 0.895 |
|  | M2 | 2.88±0.62 | 2.82±0.655 | After treatment F=7.254 | 0.000* |
|  | M3 | 2.67±0.71 | 2.67±0.71 |  |  |
|  | M4 | 2.67±0.58 | 1.67±1.53 |  |  |
|  | MR | 2.89±0.78 | 1.33±1.66 |  |  |
| pH | M1 | 3.95±1.38 | 4.08±0.37 | Before treatment F=1.153 | 0.339 |
|  | M2 | 4.49±0.51 | 4.13±0.41 | After treatment F=0.787 | 0.540 |
|  | M3 | 4.24±0.29 | 4.18±0.50 |  |  |
|  | M4 | 4.70±0.66 | 4.53±0.51 |  |  |
|  | MR | 4.44±0.51 | 4.11±0.31 |  |  |
| Cleanliness (I/II/III) | M1 | 5/4/2  (45.5/36.4/18.2%) | 7/2/2  (63.6/18.2/18.2%) | Before treatment Fisher=5.984 | 0.668 |
|  | M2 | 10/5/1  （62.5/31.3/6.3%) | 13/1/2  (81.3/6.3/12.5%) | After treatment Fisher=10.947 | 0.125 |
|  | M3 | 4/3/2  (44.4/33.3/22.2%) | 7/1/1  (77.8/11.1/11.1%) |  |  |
|  | M4 | 1/2/0  （33.3/66.7/0%） | 1/1/1  (33.3/33.3/33.3%) |  |  |
|  | MR | 7/1/1  (77.8/11.1/11.1%) | 4/5/0  (44.4/55.6/0%) |  |  |
| Leukocyte counts (<10/hp vs ≥10/hp) | M1 | 8/3  (72.7/27.3%） | 10/12/21  (90.9/9.1%） | Before treatment Fisher=4.165 | 0.381 |
|  | M2 | 13/3  (81.3/18.8%) | 14/2  (87.5/12.5%) | After treatment Fisher=1.997 | 0.846 |
|  | M3 | 8/1  (88.9/11.1%) | 8/1  (88.9/11.1%) |  |  |
|  | M4 | 1/2  （33.3/66.7%） | 2/1  （66.7/33.3%） |  |  |
|  | MR | 6/3  (66.7/33.3%) | 8/1  (88.9/11.1%) |  |  |
| Classification of *Lactobacillus* (I/IIa/IIb/III) | M1 | 0/0/7/3  (0/0/70/30%) | 1/7/3/0  (9.1/63.6/27.3/0/%) | Before treatment Fisher=1.800 | 0.801 |
|  | M2 | 0/0/8/8  (0/0/50/50%) | 0/0/7/9  (0/0/43.8/56.3%) | After treatment Fisher=29.587 | 0.000* |
|  | M3 | 0/0/4/5  (0/0/44.4/55.6%) | 0/0/5/4  (0/0/55.6/44.4%) |  |  |
|  | M4 | 0/0/2/1  （0/0/66.7/33.3%） | 0/2/1/0  （0/66.7/33.3/0%） |  |  |
|  | MR | 0/0/5/4  (0/0/55.6/44.4%) | 2/3/3/1  (22.2/33.3/33.3/11.1%) |  |  |
| *Trichomonas* (+) |  | 0 | 0 |  |  |
| Fungus (+) |  | 48 | M3=9，M4=3 | χ ^2^=48.000 | 0.000* |
| BV (Negative/intermediate/typical) | M1 | 0/4/7  (0/36.4/63.6%) | 11/0/0  (100/0/0%) | Before treatment Fisher=2.289 | 0.735 |
|  | M2 | 0/6/10  (0/37.5/62.5%) | 0/10/6  (0/62.5/37.5%） | After treatment Fisher=41.808 | 0.000* |
|  | M3 | 0/2/7  (0/22.2/77.7%) | 0/7/2  (0/77.8/22.2%) |  |  |
|  | M4 | 0/2/1  （0/66.7/33.3%） | 3/0/0 |  |  |
|  | MR | 0/4/5  (0/35.6/64.4%) | 6/2/1  (66.7/22.2/11.1%) |  |  |

**p*<0.05

**Supplementary Table 3** Characteristics of the vaginal microbiome in MR group before and after drug treatment

| Classifications | Before treatment | After treatment | After recurrence | Comparison statistic F of the 3 subgroups | *p* | χ ^2^ of the latter 2 subgroups | *p* |
| --- | --- | --- | --- | --- | --- | --- | --- |
| Amsel’s diagnostic criteria | 4.89±1.54 | 1.11±1.62 | 1.89±1.45 | 15.169 | 0.000 | T=-1.074 | 0.299 |
| Nugent score | 6.89±1.97 | 2.56±2.70 | 3.89±3.02 | 6.568 | 0.005 | -0.988 | 0.338 |
| AV score | 2.89±0.78 | 1.33±1.67 | 2.22±1.39 | 3.099 | 0.063 | -1.231 | 0.236 |
| pH | 4.44±0.51 | 4.11±0.31 | 4.23±0.41 | 1.728 | 0.199 | -0.195 | 0.848 |
| Cleanliness（I/II/III） | 7/1/1  (77.8/11.1/11.1%) | 4/5/0  (44.4/55.6/0%) | 6/3/0  (63/33.3/3.7%) | Fisher=5.185 | 0.264 | χ ^2^=0.900 | 0.343 |
| Leukocyte counts (<10/hp vs ≥10/hp) | 6/3  （66.7/33.3%)） | 8/1  (88.9/11.1%) | 8/1  (88.9/11.1%) | 1.758 | 0.567 |  |  |
| Classification of *Lactobacillus* (I/IIa/IIb/III) | 0/0/5/4  (0/0/55.6/44.4%) | 2/3/3/1  (22.2/33.3/33.3/11.1%) | 1/1/5/2  (11.1/11.1/55.6/22.2%) | 7.116 | 0.280 | Fisher=2.239 | 0.616 |
| *Trichomonas* (+) | 0 | 0 | 0 |  |  |  |  |
| Fungus (+) | 9 | 0 | 9 |  |  |  |  |
| BV (Negative/intermediate/typical) | 0/4/5  (0/44.4/55.6%) | 6/2/1  (66.7/22.2/11.1%) | 5/1/3  (55.6/11.1/33.3%) | 11.030 | 0.021 | Fisher=1.477 | 0.658 |
