# Supplementary material for: Correlation Analysis of Vaginal Microbiome Changes and Bacterial Vaginosis Plus Vulvovaginal Candidiasis Mixed Vaginitis Prognosis
Source: Front Cell Infect Microbiol. 2022 Mar 8;12:860589. doi: 10.3389/fcimb.2022.860589 (PMC8970117; doi:10.3389/fcimb.2022.860589)
Supplement: Supplementary file 2 [file DataSheet_2.pdf]

Supplementary Material

According to the abundance distribution of metabolites in vaginal secretions of subjects under different drug treatment effects, Partial Least Squares Discriminant Analysis (PLS-DA) and T-test were used to analyze the changes of key metabolites in vaginal secretions under different treatment effects.

As shown in Figure S1 (Differential metabolites between subjects in M1 and M2) and S2 (Distribution of differential metabolite abundance of subjects in M1 and M2), in group M1 and M2, Fig. S1A shows a total of 331 metabolites differed between M1.1 and M1.2. Among them, 156 metabolites were down-regulated after drug treatment (log2FC<-1 and p value<0.01 and VIP> 1), and the most significantly down-regulated metabolites (VIP>2) included (R) -3-hydroxy myristic acid, styrene, acetophenone, tyramine et al. (Fig. S2). A total of 155 metabolites were up-regulated (log2FC>1 and p value<0.01 and VIP>1) (Fig. S1A), and the most significantly up-regulated metabolite (VIP>2) was mainly maltotriose (Fig. S2). Meanwhile, Fig. S1C shows 115 metabolites of difference between M2.1 and M2.2, among which 87 metabolites were down-regulated after drug treatment (log2FC<-1 and p value<0.01 and VIP> 1), and the most significantly down-regulated metabolites (VIP>2) included 11-deoxy prostaglandin F1α, oleoyl ethanolamide etc. (Fig. S2). 28 metabolites (log2FC>1 and p value<0.01 and VIP> 1) were up-regulated and the most significantly up-regulated metabolites (VIP>2) were mainly octopine as well as N6-succinyl Adenosine (Fig. S2). On the other hand, the metabolites of M2 before and after drug treatment (M2.1&M2.2) and M1 before and after drug treatment (M1.1&M1.2) were significantly different. There were 11 differential metabolites between M2.1 and M1.1, and 99 differential metabolites between M2.2 and M1.2 (Fig. S1B, Fig. S1D and Fig. S2).

As shown in Fig. S3 (Differential metabolites among subjects in M1 and M3), there were no significant differences in metabolites of group M3 before and after drug treatment (M3.2 vs M3.1), suggesting that the drug treatment did not cause significant changes in overall metabolism. On the other hand, metabolites before and after drug treatment in M3 (M3.1&M3.2) were significantly different from those in M1 (M1.1&M1.2). There were 11 differential metabolites between M1.1 and M3.1 (log2FC<-1 or log2FC>1 and p value<0.01 and VIP >1), and 133 differential metabolites between M3.2 and M1.2 (Fig. S3A and Fig. S3B). Fig. S3C shows the abundance distribution of metabolites (log2FC<-1 or log2FC >1 and p value<0.01 and VIP >2) with significant differences in each group. Several metabolites up-regulated or down-regulated in the cured group (M1.2) did not change significantly in the untreated group (M3.2), including 2-hydroxyvaleric acid down-regulated significantly and 2-methoxyestradiol (2-meoE2) up-regulated significantly in the cured group. No significant changes of these metabolites were observed in the uncured group before and after treatment (M3.1 & M3.2), suggesting that drug treatment did not cause significant changes in key metabolites in the uncured group (M3).

As shown in Fig. S4 (Differential metabolites between subjects in M1 and MR) and Fig. S5 (Distribution of differential metabolite abundance of subjects in M1 and MR), 177 metabolites of the recurrenc group were different after (MR.2) and before drug treatment (MR.1) (Fig. S4A). After drug treatment, 32 metabolites were down-regulated (log2FC<-1 and p value<0.01 and VIP> 1), and the most significantly down-regulated metabolite (VIP>2) was propionyl-L-carnitine. Among 145 upregulated metabolites (log2FC>1 and p value<0.01 and VIP >1), the metabolites with the most significant up-regulation (VIP>2) were LPE, glycerophospho-n-palmitoyl, 1-Palmitoylol, DY131. Meanwhile, 6 metabolites were significantly down-

regulated after recurrence (MR.3) compared with that after drug treatment (MR.2), mainly 1-palmitoylglycerol and ophospho-n-Palmitoyl. 11 metabolites were significantly down-regulated and 23 metabolites were up-regulated compared with that before drug treatment (MR.1), suggesting that the accumulation of such metabolites may be associated with the recurrence of the disease (Fig. S4C, E). On the other hand, there was no significant difference in metabolites before and after treatment in the recurrence group (MR.1&MR.2&MR.3) compared with that in the completely cured group (M1.1&M1.2), among which 16 metabolites were mainly upregulated and 27 metabolites were down-regulated in the recurrence group (Fig. S4B, D, F).
