# Supplementary figures and images for: Correlation Analysis of Vaginal Microbiome Changes and Bacterial Vaginosis Plus Vulvovaginal Candidiasis Mixed Vaginitis Prognosis
Source: Front Cell Infect Microbiol. 2022 Mar 8;12:860589. doi: 10.3389/fcimb.2022.860589 (PMC8970117; doi:10.3389/fcimb.2022.860589)

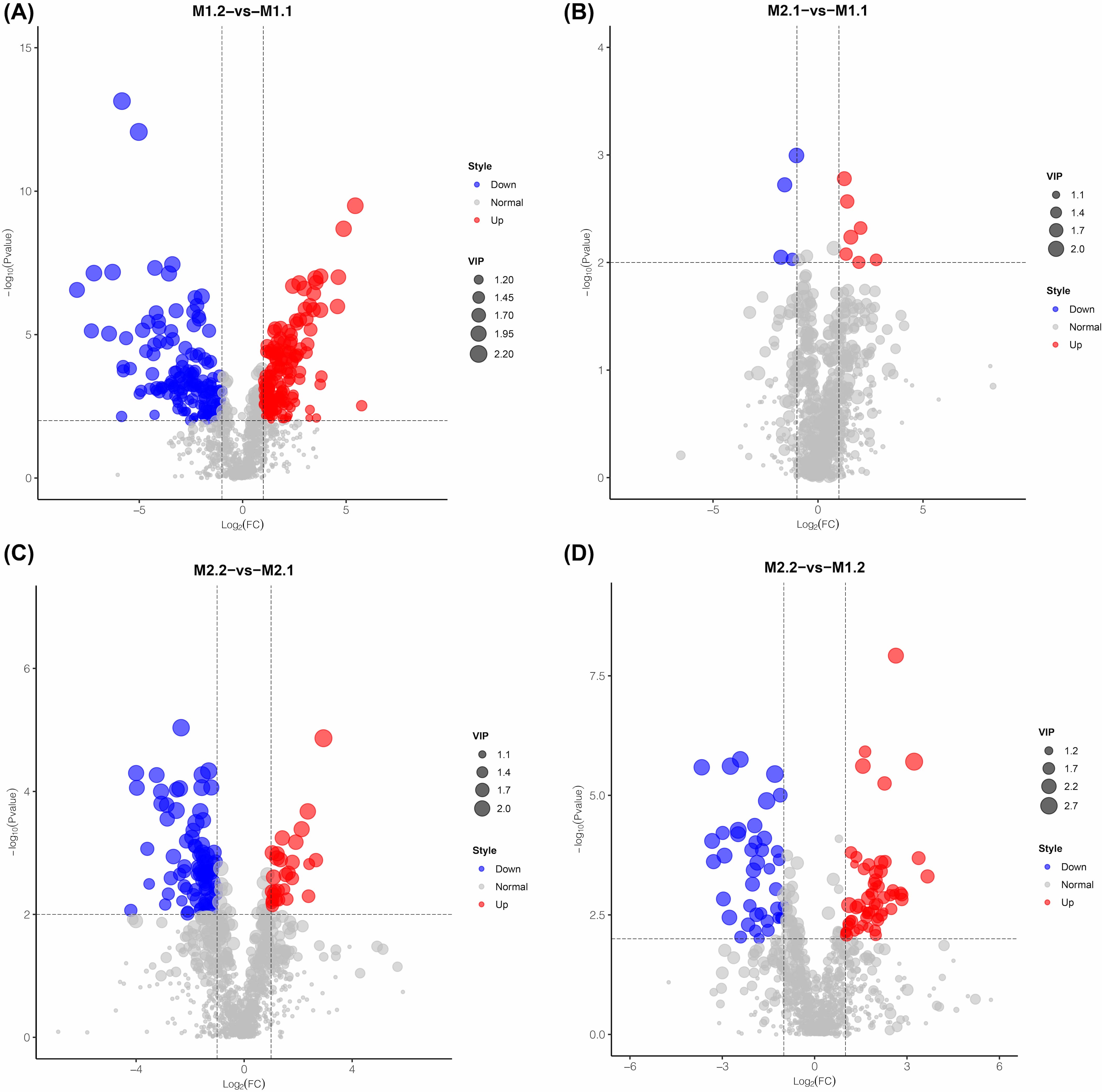

Supplement: Supplementary file 3 [file Image_1.jpeg]

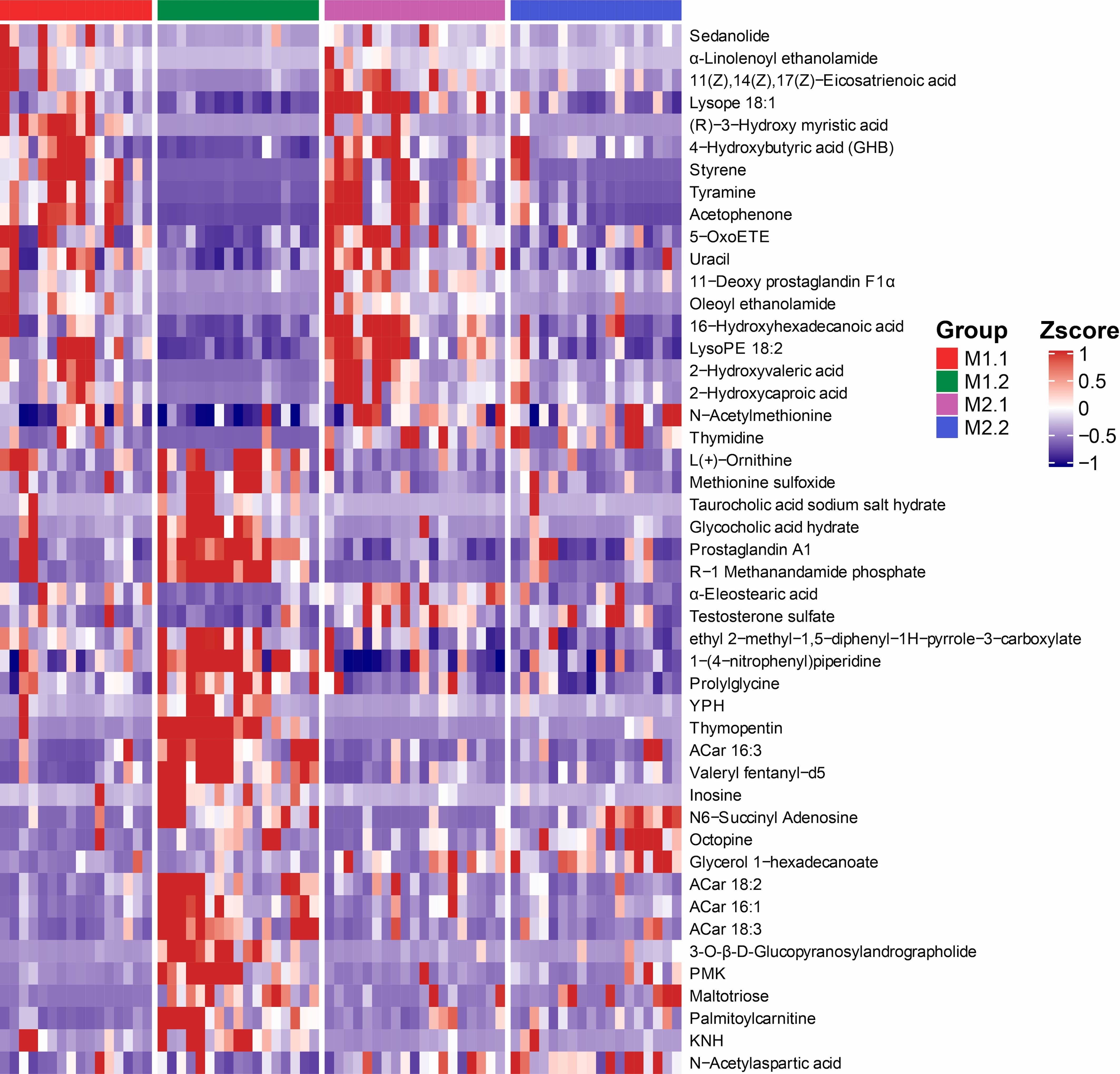

Supplement: Supplementary file 4 [file Image_2.jpeg]

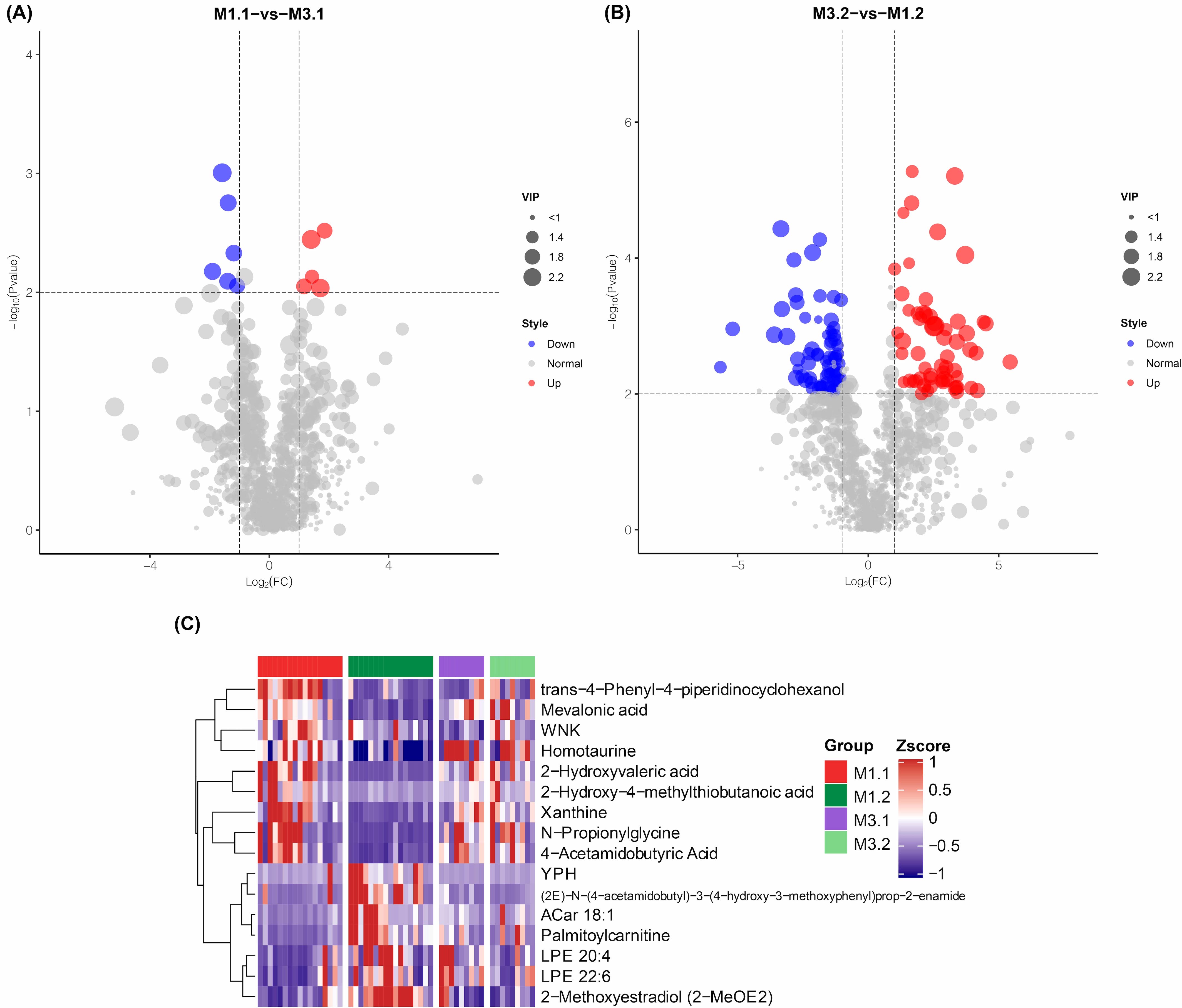

Supplement: Supplementary file 5 [file Image_3.jpeg]

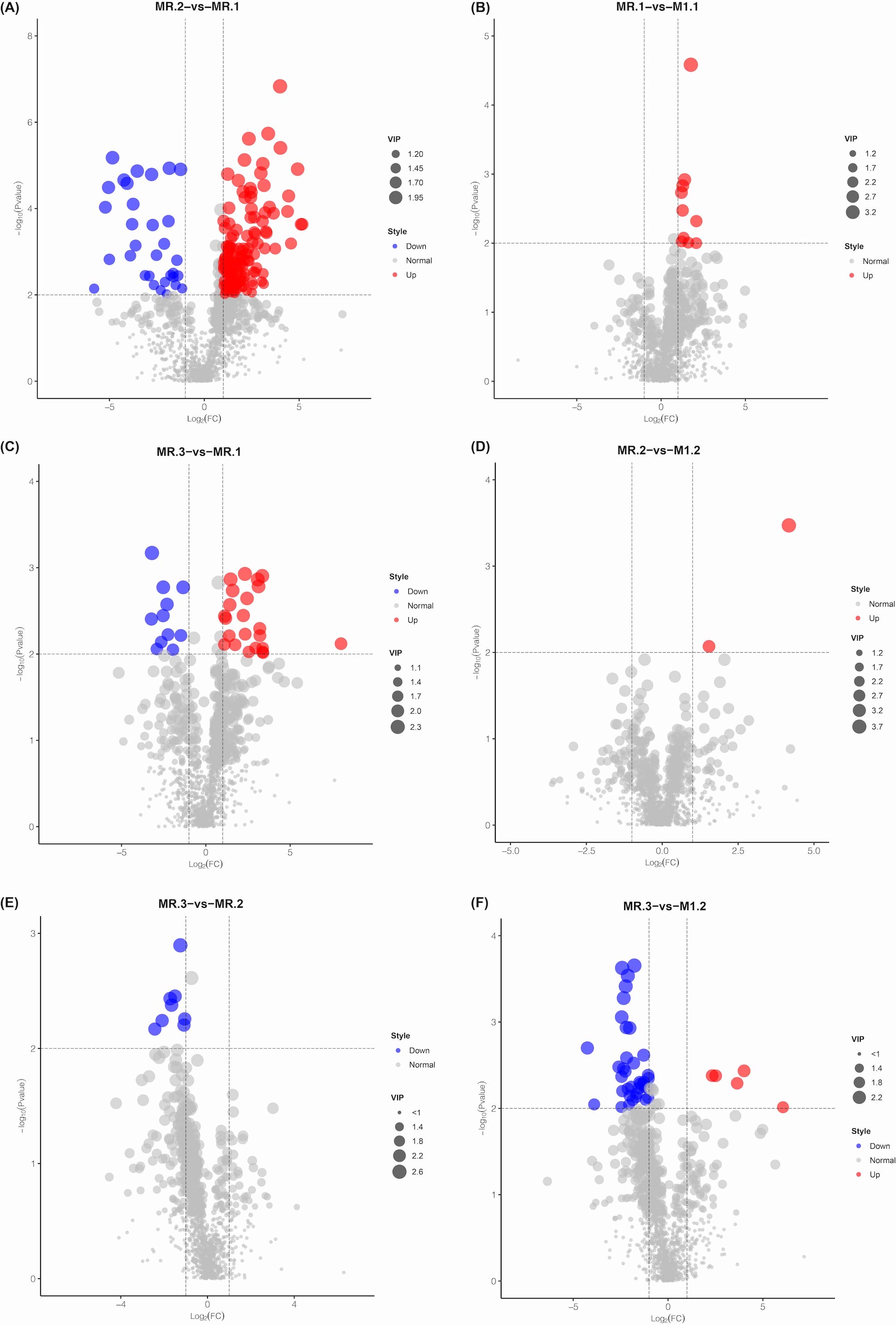

Supplement: Supplementary file 6 [file Image_4.jpeg]

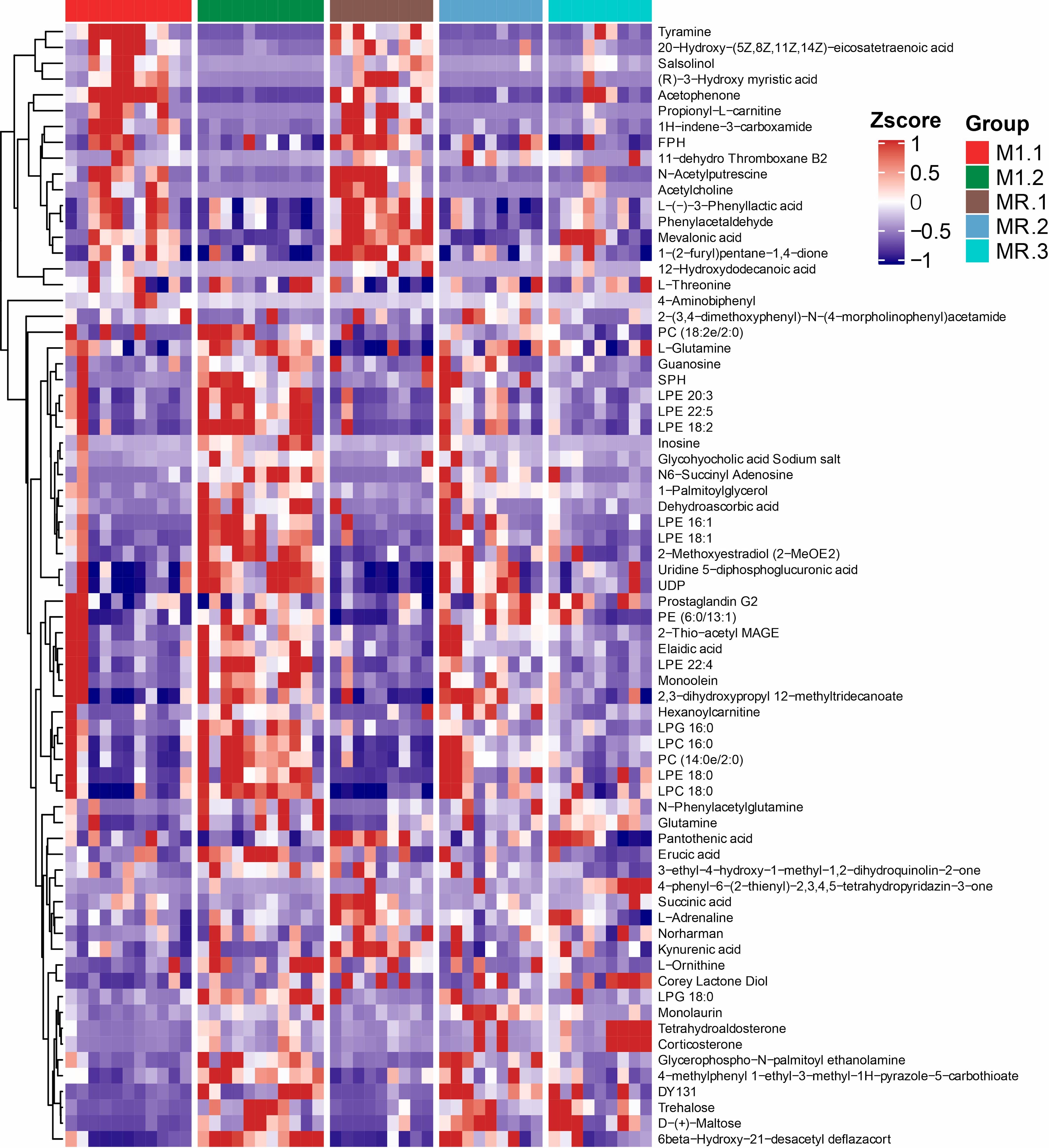

Supplement: Supplementary file 7 [file Image_5.jpeg]
